# Early experience with percutaneous photodynamic nails for sacral metastatic disease and insufficiency fractures: a retrospective cohort analysis of functionality and pain relief

**DOI:** 10.1186/s12891-025-08707-8

**Published:** 2025-05-10

**Authors:** Marilee J. Clunk, Marcos R. Gonzalez, Sonia E. Ubong, Alisha Sodhi, Joseph O. Werenski, Hayley M. Denwood, Daniel G. Tobert, Santiago A. Lozano-Calderon

**Affiliations:** 1 Musculoskeletal Oncology Service, Department of Orthopaedic Surgery, Massachusetts General Hospital, Yawkey Building, Suite 3B, 55 Fruit St, Boston, MA 02114 USA; 2 University of Toledo College of Medicine and Life Sciences, 3000 Arlington Ave., Toledo, OH 43614 USA; 3 Department of Surgery, University of Cambridge, Cambridge, UK; 4 Boston University Chobanian and Avedisian School of Medicine, 72 East Concord St, Boston, MA 02118 USA; 5 Orthopaedic Spine Service, Department of Orthopaedic Surgery, Massachusetts General Hospital, 55 Fruit St, Boston, MA 02114 USA

**Keywords:** Pathologic fracture, Metastatic bone disease, Percutaneous

## Abstract

**Background:**

Treatment of impending pathologic fractures and insufficiency fractures of the sacrum due to metastatic disease and radiation is challenging. The use of traditional hardware is limited by poor bone quality and presence of non-contained defects. The role of minimally invasive photodynamic nails (PDN) for treatment of these fractures remains poorly understood.

**Methods:**

Patients with symptomatic impending pathologic fractures of the sacrum due to metastatic bone disease, multiple myeloma, or insufficiency fractures from radiation osteitis who underwent PDN stabilization were identified. Primary outcomes included post-operative complications, pain relief, opioid consumption, and function. Pain was assessed using Visual Analog Scale (VAS), and function was measured using the Combined Pain and Ambulatory Function (CPAF) score. Outcomes were assessed preoperatively at 6 weeks, 3 months, 6 months, 1 year and 2 years.

**Results:**

Fourteen patients (median age 70, 50% female) underwent sacral PDN stabilization between 2020 and 2023, with a median 1.4-year follow-up. Overall complication rate was 7% (1/14 patients), with one case of venous thromboembolism. Median pain VAS decreased from 7 preoperatively to 6 at 6 weeks (*p* = 0.02), and to 4 at 2 years (*p* = 0.002). Median CPAF score improved from 6 preoperatively to 7 at 3 months and remained at this level through the 2-year follow-up. Chronic opioid use decreased from 85.7% preoperatively to 60% at 2 years.

**Conclusion:**

PDN stabilization sacral insufficiency fractures in oncologic patients is a safe surgical technique that effectively restores patient ambulatory function and provides rapid pain relief. Further research with larger cohorts is warranted to confirm these promising results.

**Level of evidence:**

III.

**Clinical trial number:**

Not applicable.

## Introduction

Sacral metastases are increasingly prevalent due to improved oncological therapies extending survival and an aging population reaching metastatic stages [1–4]. This demographic shift has been accompanied by an increased prevalence of compromised bone integrity due to (1) progressive lytic lesions, (2) radiation-induced osteonecrosis from pelvic radiotherapy, and (3) systemic effects, including chemotherapy-induced and paraneoplastic osteoporosis. Clinically, these fractures manifest as acute-onset, severe, diffuse sacral pain and tenderness, often radiating to the hips, buttocks, and groin, with symptoms characteristically exacerbating under axial loading [5–7]. Due to the complex anatomy of the sacrum and its critical role in load-bearing and pelvic stability, treatment of these fractures is extremely challenging in the oncologic population [8, 9].

Historically, impending or insufficiency sacral fractures have been treated with open reduction and internal fixation. However, this method has largely fallen out of favor due to poor functional outcomes and a high risk of postoperative complications, including infection and implant loosening [10, 11]. Moreover, these invasive procedures often lead to delays or interruptions in systemic treatment and radiation therapy, potentially compromising overall oncological care [12]. In recent years, minimally invasive techniques have gained traction in the treatment of pathological fractures, offering potential advantages in terms of reduced surgical morbidity and faster recovery times [13–15]. Nowadays, patients are more often treated with closed reduction and percutaneous fixation with screws with or without cementoplasty. Among these innovative approaches, the use of percutaneous photodynamic nails (PDNs) has emerged as a promising option for managing impending pathologic and insufficiency sacral fractures [14, 16, 17]. PDNs are a minimally invasive percutaneous technology consisting of patient-conforming, light-sensitive polymer implants. This technique aims to address the limitations of traditional rigid and radiopaque implants while providing effective stabilization and symptom relief. The lack of metal scattering on computed tomography (CT) and magnetic resonance imaging (MRI) the possibility to add hardware to it makes this type of implant appealing for use in the oncologic population.

The purpose of the present study was to retrospectively describe our experience with percutaneous PDNs in managing impending pathological and insufficiency sacral fractures, evaluating outcomes in functionality, pain relief, complications, and local tumor control.

## Materials and methods

### Patient selection

After obtaining Institutional Review Board approval (ID: 2021P001624), we conducted a retrospective cohort study at our institution examining cases of impending pathologic or insufficiency sacral fractures treated with percutaneous PDNs between 2020 and 2023. The study included 14 consecutive adult patients (≥ 18 years old) with an oncologic history who underwent this procedure during the specified period (Table 1). Inclusion criteria encompassed (1) radiographic evidence of sacral insufficiency fracture or impending pathologic fracture confirmed by CT or MRI; (2) intractable sacral pain or referable radicular pain mechanical in nature and exacerbated by movement; (3) inability to bear weight or need for assistive devices due to sacral instability and decreased mobility directly attributable to sacral pain. During the study period, patients with complete pathologic sacral fractures were treated with other stabilization methods such as sacroiliac screws with cement augmentation and were not included in this analysis. Impending pathologic sacral fractures were defined as painful lesions with greater than 50% cortical bone destruction, or a permeative pattern over a length of bone greater than 2 cortical diameters. Exclusion criteria were the following: (1) primary indication for surgery was infection, trauma, hardware failure, or wound dehiscence; (2) unretrievable data for any of the variables outlined below. Particular attention was paid to complete follow-up data, as assessment of longitudinal outcomes was the primary focus of this study.

**Table 1:** Baseline characteristics of included patients

	Total (*N* = 14)
**Age (years)** ^**+**^	70 (62, 73)
**Female sex**	7 (50%)
**Age-adjusted CCI** ^**+**^	9.5 (9, 11)
**Primary cancer**	
Multiple myeloma	2 (14%)
Breast	2 (14%)
Lung	1 (7%)
RCC	1 (7%)
Thyroid	2 (14%)
Prostate	2 (14%)
Other	4 (29%)
**Katagiri classification**	
Slow growth	5 (36%)
Moderate growth	5 (36%)
Rapid growth	4 (29%)
**Additional sites of metastases**	9 (64%)
Lung	5 (36%)
Lymph nodes	4 (29%)
Liver	2 (14%)
Other	1 (7%)
**Systemic therapy**	12 (86%)
Neoadjuvant	9 (82%)
Adjuvant	11 (92%)
**Radiation therapy**	8 (57%)
Neoadjuvant	7 (88%)
Adjuvant	5 (63%)
**Follow-up (years)** ^**+**^	1.4 (1.2, 2.8)

*CCI*: Charlson comorbidity index; *RCC*: renal cell carcinoma

^+^ Values in these rows refer to the median and interquartile ranges between parentheses

### Variables and outcomes of interest

Patient demographic information was obtained, including age, sex, body mass index (BMI), and age-adjusted Charlson Comorbidity Index (CCI). Clinical data captured encompassed symptomatology, functional status, American Society of Anesthesiologists (ASA) classification, primary cancer type, Katagiri classification, metastatic burden, and comprehensive treatment history including surgical, radiotherapeutic, and systemic therapy interventions. Surgical variables collected included the number of PDNs employed, procedure type (categorized as simple or complex), use of additional hardware, operative time, estimated blood loss, and application of intraoperative ablation. Length of stay was also recorded for each patient (Table 2). Simple procedures involved using 1 or 2 PDNs alone for sacral stabilization, whereas complex procedures required additional hardware, such as total hip arthroplasty or intramedullary nails. Additional hardware was typically necessary due to diffuse metastatic disease and the need to stabilize additional bone structures.

**Table 2:** Surgical characteristics and post-operative complications

	Total (*N* = 14)	Simple (*N* = 7)	Complex (*N* = 7)	*p*-value
**PDN (n)**				0.58
1	9 (64%)	4 (57%)	5 (71%)	
2	5 (36%)	3 (43%)	2 (29%)	
**Operative time (min)** ^**+**^	201 (124, 315)	124 (98, 187)	315 (215, 425)	**0.009**
**EBL (mL)** ^**+**^	225 (50, 500)	50 (50, 150)	500 (300, 650)	0.052
**Intraoperative ablation**	2 (15%)	1 (14%)	1 (17%)	0.91
**LOS (days)** ^**+**^	2.5 (1, 7)	1 (1, 6)	4 (2, 8)	0.14
**ICU admission**	0 (0%)	0 (0%)	0 (0%)	
**Complications**				
PDN failure	0 (0%)	0 (0%)	0 (0%)	-
VTE	1 (7%)	1 (14%)	0 (0%)	0.30
Reoperation	0 (0%)	0 (0%)	0 (0%)	-
Readmission	0 (0%)	0 (0%)	0 (0%)	-

*EBL*: estimated blood loss; *ICU*: intensive care unit; *LOS*: length of stay; *PDN*: photodynamic nail; *VTE*: venous thromboembolism

^+^ Values in these rows refer to the median and interquartile ranges between parentheses

The primary outcomes of interest were post-operative complications, pain relief, opioid consumption, and patient function. Post-operative complications included implant failure, venous thromboembolism (VTE), reoperation, and readmission rates. Pain relief was assessed using the Visual Analog Scale (VAS), which was retrospectively collected through systematic chart review of clinical documentation at each follow-up visit. Patient function was measured using the Combined Pain and Ambulatory Function (CPAF) score. Opioid use was evaluated at each timepoints based on the prescriptions issued by our institution and filled by patients, as well as patient charts. Additionally, changes in Eastern Cooperative Oncology Group Performance Status (ECOG-PS) were evaluated to assess overall functional outcomes. The ECOG Performance Status scale was the only physician-based tool employed. ECOG scores range from 0 to 4, with 0 denoting full activity and 4 indicating complete disability.

### Surgical technique

All procedures were performed for symptomatic impending pathologic or insufficiency sacral fractures. Patients were positioned prone on a radiolucent table after anesthetic induction. Prior to incision, the surgical site was confirmed using intraoperative navigation and/or fluoroscopy, with traditional lateral, inlet, and outlet views of the sacrum. Intra-operative navigation was preferred due to the poor bone quality and complex lesions typical of tumor-associated sacral fractures, which often diminish haptic feedback during drill progression. Following site confirmation, a lateral incision was made along the plane posterior to the greater tuberosity in the proximal third of the gluteal area. A drill was advanced through the iliac bones into the sacrum, followed by a straight awl to expand the pathway. The drill was then carefully exchanged for 2.0 mm ball-tipped (3.0 mm) guidewire. The appropriate PDN length was determined using a measurement device or an additional wire. The interosseous space was sequentially reamed from 6 mm to 8 mm in 0.5 mm increments.

The PDN was deployed under fluoroscopic guidance and cured using visible light, providing a customized intramedullary implant for sacral fracture stabilization. We typically inflate the balloons to their maximum recommended volume, with careful fluoroscopic monitoring to avoid over-distension. In most cases, the PDN spans all four iliac cortices to provide maximum stability.

**Fig. 1:**
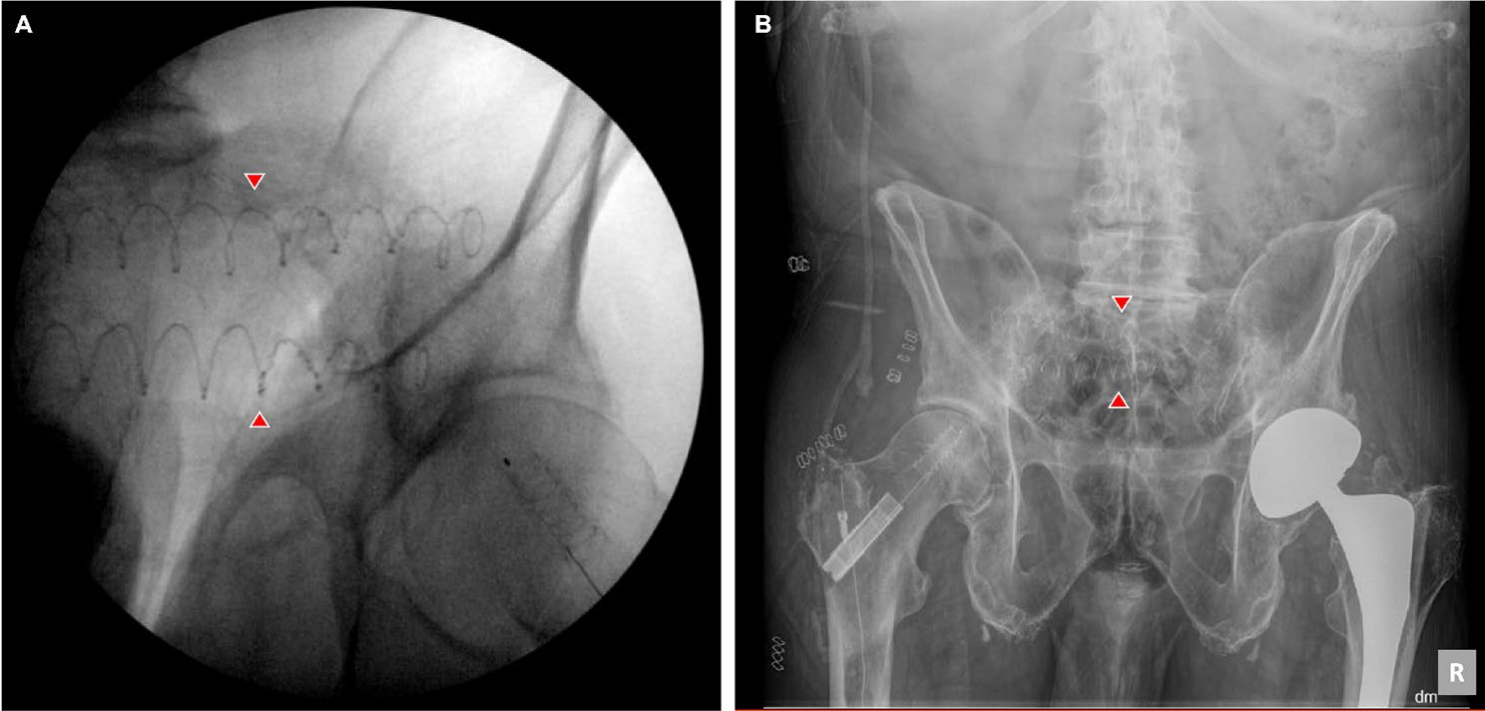
(**A**) Intraoperative fluoroscopy, and (**B**) postoperative AP radiograph showing adequate placement of 2 photodynamic nails

**Fig. 2:**
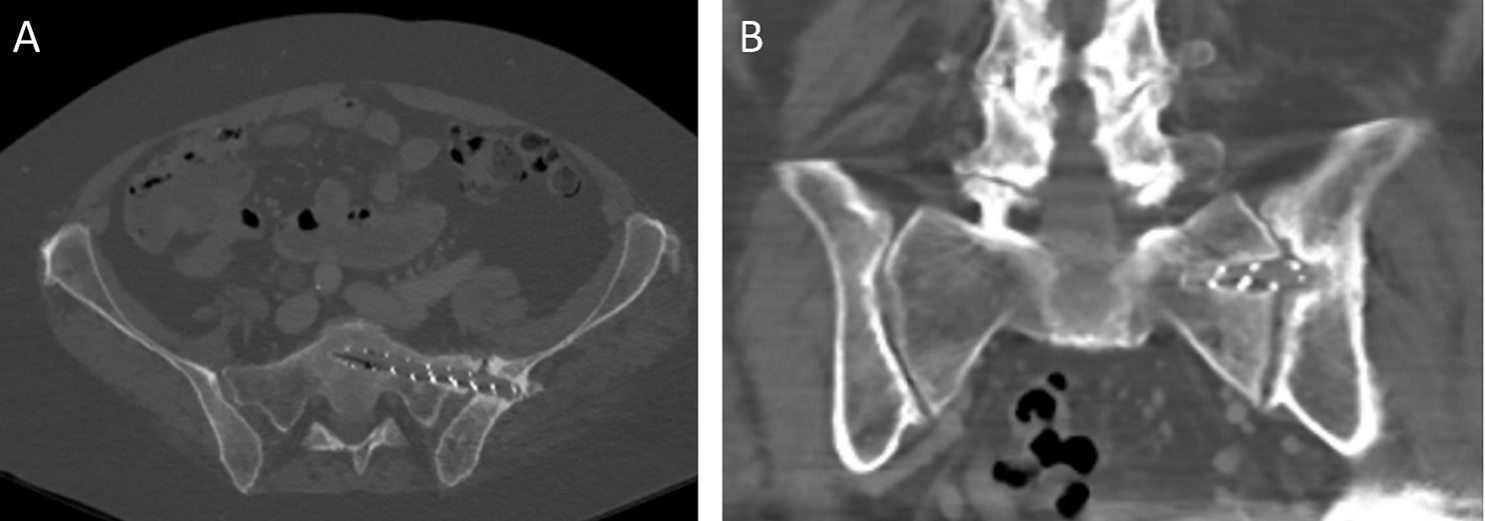
Post-operative CT scan showing adequate positioning of the photodynamic nail from the left iliac bone into the S1 vertebral body on (**A**) axial and (**B**) coronal planes

Adequate positioning of the PDNs on intraoperative fluoroscopy and postoperative radiographs can be visualized on Fig. 1. Similarly, axial and coronal slices of CT scan demonstrating adequate positioning of the PDN for sacral stabilization can be seen on Fig. 2.

### Statistical analysis

Demographic, clinical, and treatment characteristics are reported using descriptive statistics. The Shapiro-Wilk test was employed to evaluate normality of the distribution of data. Due to a non-normal distribution, median and interquartile ranges were reported for continuous variables. The Kruskal-Wallis test was used to compared continuous variables at different timepoints. A post-hoc nonparametric comparison of subgroups was conducted using the Dunn test. A p value of < 0.05 was considered significant. All statistical analyses were conducted using Stata (StataCorp).

## Results

### Demographics, operative and oncologic data

Between 2020 and 2023, 14 patients aged 57 to 76 years (median age, 70 years; 50% male) underwent percutaneous PDN stabilization for impending pathologic or insufficiency sacral fractures (Table 1). Of the 14 patients in our cohort, 11 (79%) had impending pathologic fractures due to metastatic disease or multiple myeloma, while only 3 (21%) had insufficiency fractures from radiation osteitis. Median age-adjusted CCI was 9.5, and the most common types of primary cancers were multiple myeloma (14%) and breast carcinoma (14%). Systemic therapy and radiation therapy was administered to 86% and 57% of patients, respectively. Among patients who received radiation therapy, all had radiation fields that included the sacrum. The median follow-up time was 1.4 years.

Operatively, 64% of patients had a single PDN placed, while 36% had two PDNs at two different sacral levels. The median operative time was 201 min, with a significant difference between simple (median, 124 min) and complex (median, 315 min) procedures (*p* = 0.009) (Table 2). Median estimated blood loss was 225 mL and intraoperative ablation was performed in 15% of cases. Median length of stay was 2.5 days, with no significant difference between simple and complex procedures (*p* = 0.14). The individual characteristics of each case are available on (Table 3).

**Table 3:** Individual characteristics of included patients

*N*	Sex	Age	AA-CCI	Primary tumor	Ablation	Procedure type	Additional hardware	Operative time (min)	EBL (mL)	Reoperation	Post-operative complication	FU (months)
1	M	74	10	Prostate	Yes	Simple		124	50	No	No	5.3
2	F	76	11	Melanoma	No	Complex	THA	315	300	No	No	44.6
3	F	57	4	Multiple myeloma	No	Simple		87	50	No	No	38.1
4	M	73	10	Prostate	No	Complex	External fixator	361	500	No	No	37.4
5	M	62	5	Multiple myeloma	No	Simple		284	150	No	No	3
6	M	72	10	Pharyngeal SCC	No	Simple		187	800	No	No	14.4
7	F	69	9	Endometrioid adenocarcinoma	No	Complex	Spine rod	215	650	No	No	14.5
8	F	65	9	Breast	No	Complex	THA + periacetabular PDNs	425	350	No	No	15.1
9	M	73	12	RCC	No	Complex	Femur IMN	219	500	No	No	17
10	M	70	12	Hepatocellular	No	Complex	THA + periacetabular PDNs	435	900	No	No	15.6
11	F	57	9	Lung	No	Simple		119	50	No	No	19.4
12	F	59	2	Thyroid	Yes	Complex	Periacetabular PDNs	174	50	No	No	10.6
13	F	68	9	Breast	No	Simple		156	150	No	No	22.5
14	M	70	11	Thyroid	No	Simple		98	25	No	No	33.5

*AA-CCI*: age-adjusted Charlson comorbidity index; *EBL*: estimated blood loss; *FU*: follow-up; *IMN*: intramedullary nail; *PDN*: photodynamic nail; *RCC*: renal cell carcinoma; *SCC*: squamous cell carcinoma; *THA*: total hip arthroplasty

### Complications

There were no instances of percutaneous PDN failure, reoperation, or readmission. One patient (7%) developed deep vein thrombosis (DVT) during the post-operative course (Table 2). No patients required ICU admission.

### Pain and ambulatory function

**Fig. 3:**
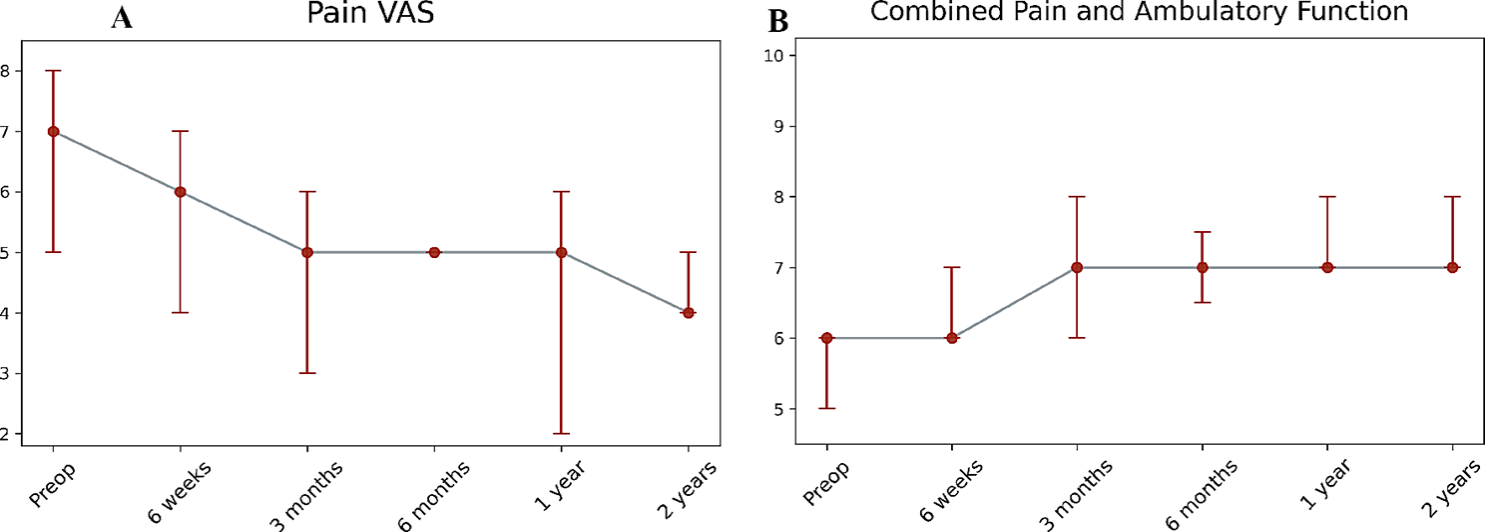
Pain and functional outcomes using the (**A**) pain visual analog scale, and (**B**) the Combined Pain and Ambulatory Function

The median pain VAS score decreased from 7 preoperatively to 6 at 6 weeks postoperatively (*p* = 0.02). Further reductions were observed at subsequent timepoints, with median scores of 5 at both 3 and 6 months, and 4 at 2 years post-intervention. All postoperative time points showed statistically significant improvements compared to baseline (*p* < 0.05 for all comparisons) (Fig. 3A). The CPAF score improved from a preoperative score of 6 to 7 at the 3-month mark (*p* = 0.009). This improved score was maintained through the 2-year follow-up period (Fig. 3B).

Subgroup analysis revealed that patients undergoing simple procedures (*n* = 7) achieved greater VAS pain score reduction at 6 weeks compared to those with complex procedures (*n* = 7) (median reduction, 2 vs. 1, *p* = 0.04). However, CPAF scores at 1 year showed no significant difference between these groups (*p* = 0.38).

### Opioid consumption

**Fig. 4:**
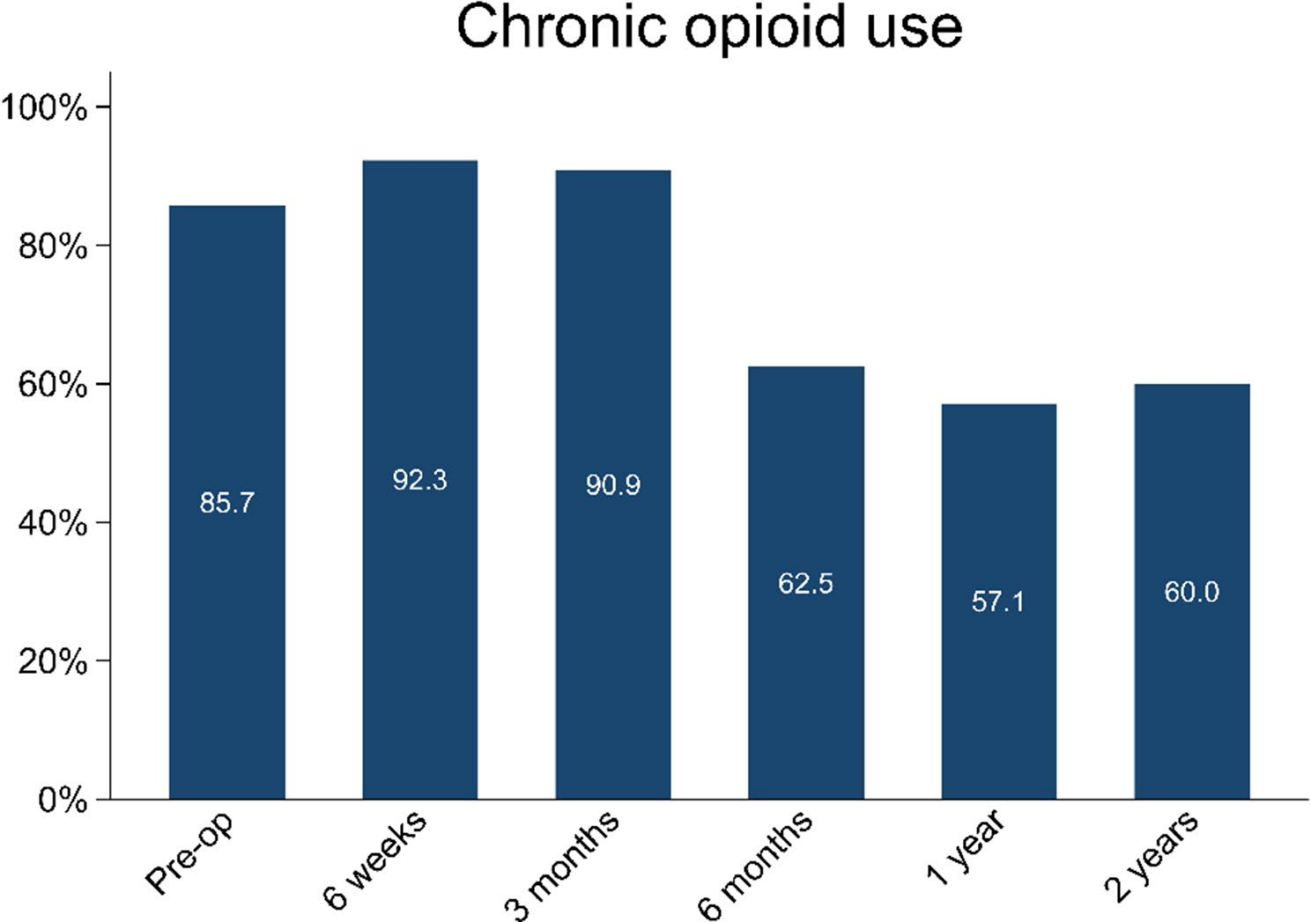
Percentage of patients prescribed opioid analgesia at different time points

Chronic use of opioids decreased from 85.7% preoperatively to 62.5% at the 6-month postoperative mark (*p* = 0.10) (Fig. 4). At the 1- and 2-year postoperative timepoints, opioid consumption occurred in 57.1% and 60% of patients, respectively.

### ECOG scores

**Fig. 5:**
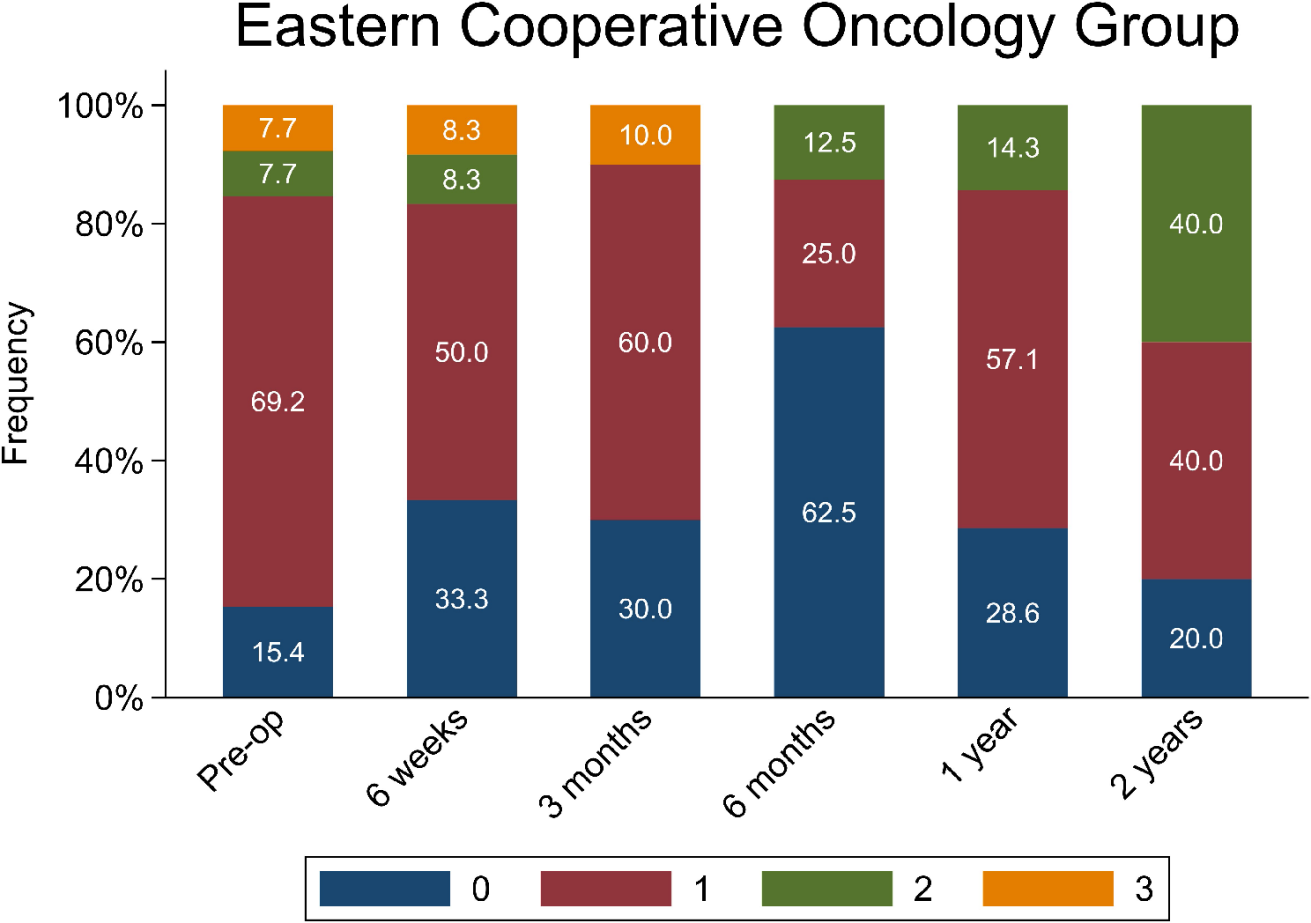
Distribution of patient scores on the Eastern Cooperative Oncology Group (ECOG) Performance Status Scale at different time points

Preoperatively, 84.6% of patients had an ECOG score between 1 and 3, and only 15.4% had a score of 0. By 6 months postoperatively, the percentage of patients with an ECOG score of 0 increased to 62.5% and only 37.5% had scores between 1 and 2 (Fig. 5).

## Discussion

Pathological fractures in oncologic patients often signify end-stage disease, with approximately 50% of patients not surviving beyond 6 months following surgical intervention and only a small fraction surviving for several years [18, 19]. In this context, preventing pathological fractures becomes paramount, particularly in weight-bearing areas such as the sacrum. This study represents the first comprehensive analysis of the percutaneous PDN for tumor-associated sacral impending pathologic fractures and insufficiency fractures, addressing a critical gap in the literature.

Sacral fixation interventions, while effective, are associated with significant post-operative complications. Percutaneous sacroiliac screw fixation, a common approach for sacral insufficiency fractures, has shown notable complication rates. Eckardt et al. reported a 20% complication rate in 50 patients with fragility fractures of the pelvis treated with percutaneous screw fixation, including screw malpositioning, secondary fracture displacement, and need for revision surgery [20]. Kramer et al.’s study on 3D-navigated screw fixation for sacral fragility fractures reported a screw loosening rate of 19% with a significant portion requiring re-operation (11%) [21]. While these studies focused on patients with longer life expectancies than our oncologic cohort—providing extended periods for hardware failures to develop—they nonetheless highlight important risks of percutaneous sacral techniques that remain relevant across patient populations. Moreover, these procedures often result in prolonged hospital stays and delayed mobilization, increasing the risk of thromboembolic events and respiratory complications [22, 23]. The challenges are exacerbated due to substantial weight-bearing loads, potential skin toxicity from radiation therapy in oncologic patients, decubitus pressure, and the presence of critical neurovascular structures. These factors, combined with the high comorbidity burden in our patient population (median age-adjusted CCI of 9), underscore the need for less invasive alternatives with potentially lower complication rates.

PDNs have demonstrated promising results in various fracture areas, including long bones and pelvis, offering improved stability with minimal surgical trauma. The radiolucent properties of PDNs allow for better monitoring of tumor response to radiation therapy, while their flexible nature may better accommodate the complex biomechanics and anatomy of the sacrum. Furthermore, the minimally invasive insertion technique could potentially reduce post-operative complications and allow for earlier resumption of systemic treatments and radiation therapy. Given these advantages, exploring the use of PDNs for weight-bearing applications in the sacrum is important, potentially offering a valuable option for patients who are poor candidates for more invasive procedures.

The complication profile observed in our cohort underscores the safety of this technique for sacral insufficiency fractures. Our series demonstrated a low overall complication rate (7% [1/14]), with the single adverse event being a venous thromboembolism. This rate compares favorably with those reported in previous studies of minimally invasive sacral fixation techniques. For instance, Cofano et al., in their systematic review of sacroiliac screw fixation in spine metastatic disease, reported complication rates ranging from 0 to 41.7%, with screw loosening or pullout (20%) and neurological deficits (13.3%) being the most common complications [24]. The absence of implant-related complications in our series represents a promising finding that may reflect the unique advantages of PDNs. The customizable nature of PDNs allows for adaptation to individual pelvic anatomy and potentially reduces the risk of mechanical failure. While our outcomes are encouraging, we acknowledge that our findings should be interpreted in the context of our cohort’s characteristics, including the small sample size and the relatively shorter survival times typical of oncologic patients compared to those with fragility fractures. The occurrence of VTE in one patient (7%) warrants consideration in the context of the inherently elevated thromboembolic risk in cancer patients. A meta-analysis by Horsted et al. reported VTE incidence rates of 7% in cancer patients undergoing surgery, suggesting that our observed rate is consistent with the background risk in this population [25]. Nevertheless, this finding underscores the importance of appropriate thromboprophylaxis in this high-risk cohort. Infection, often cited as a significant concern in sacral fracture fixation, was notably absent in our series. This contrasts sharply with reported infection rates of up to 33% in traditional open sacral plating procedures [26].

Our findings demonstrated improvements in both pain and function after percutaneous PDN fixation. Median VAS pain scores decreased from 7 preoperatively to 6 at 6 weeks, while CPAF scores improved from 6 preoperatively to 7 at 3 months, with improvements stable up to 2 years. The gradual improvement in pain scores over time rather than immediate substantial relief likely reflects the progressive stabilization of the sacrum as the surrounding soft tissues heal. We typically counsel patients to expect modest initial pain improvements (1–2 points on the VAS scale) in the first 6 weeks, with continued improvement over the subsequent 6 months as bony healing and tissue remodeling occur. Additionally, in our oncologic population, pain improvement may be influenced by concurrent treatments such as radiation therapy or systemic therapies that address the underlying disease process.

This study evaluated both measures at multiple time points over a median 1.4-year follow-up, addressing limitations in current literature. A recent systematic review revealed that studies examining surgical fixation of sacral fractures often had limited pain assessment points, with only 4 out of 10 papers reporting VAS scores at two separate time points postoperatively [27]. Similarly, functional outcome reporting in existing literature is often incomplete or lacking in detail. For instance, Wahnert et al. stated patients that regained their pre-operative mobility level without specifying the timeline for this recovery [28]. Hartensuer et al. provided more detail, noting that 52% of patients mobilized with a frame, 29.73% with crutches, and 8.79% could not be mobilized before discharge [24]. These variations in reporting highlight the need for more standardized and comprehensive assessment of functional outcomes in sacral fracture management research. Our approach of assessing both VAS and CPAF scores up to 2 years post-intervention offers a more detailed picture of long-term outcomes.

Use of PDN stabilization also reduced chronic opioid use, decreasing from 85.7% preoperatively to 60% at 2 years post-intervention. sustained decrease suggests that PDN fixation contributes to long-term improvements in pain management, potentially enhancing quality of life and mitigating opioid-related complications in this high-risk population [29]. Furthermore, reduced opioid use may benefit oncologic treatment, as opioids have been associated with decreased efficacy of certain chemotherapeutic agents [30, 31].

One potential advantage of PDNs that warrants further investigation is the reduced risk of neurological complications. Unlike cement augmentation, which carries risks of extravasation into neural foramina, or traditional hardware that may back out and impinge on nerve roots, PDNs can be expanded under fluoroscopic control with real-time visualization of implant boundaries. While our study did not specifically track minor neurological symptoms, no major neurological complications were documented during follow-up, suggesting that PDNs may offer favorable safety profiles regarding nerve preservation. Future studies with specific neurological assessments would help confirm this potential advantage.

Based on our experience with PDNs in this cohort, the decision between using PDNs versus screws with cement augmentation is primarily based on lesion characteristics and surgeon preference. PDNs are particularly favored for patients with poor bone quality, where screw purchase may be suboptimal, and for lesions with complex geometries that may not be adequately addressed with rigid implants. PDNs are especially effective for uncontained defects in the sacrum, offering reliable stability even when cortical boundaries are compromised, which represents a significant advantage in these challenging cases. Additionally, PDNs are preferred when post-operative imaging surveillance of tumor response is anticipated, as their radiolucent properties minimize artifact on CT and MRI. Conversely, sacroiliac screws with cement augmentation are typically chosen for complete pathologic fractures or cases where immediate stability is critical.

Our study is not exempt of limitations. The relatively small sample size (*n* = 14) and single-center design limit generalizability and preclude robust subgroup analyses. The median follow-up of 1.4 years, though longer than in most studies in metastatic bone disease, may not fully elucidate long-term implant-related issues or late oncological complications. Nonetheless, the study has several merits. The comprehensive assessment of functional parameters, including VAS and CPAF scores, provides a multidimensional view of patient outcomes. Importantly, the longitudinal data collection at several time points up to two years post-intervention offers insights into the durability of treatment effects, surpassing the follow-up duration of many previous studies in this field. Future research should address these limitations through multicenter studies with larger cohorts and extended follow-up periods to further evaluate the long-term efficacy and safety profile of PDN fixation in this patient population.

## Conclusion

Percutaneous stabilization of sacral impending pathologic fractures or insufficiency fractures with PDNs is a low-risk procedure that offers swift pain relief and significant improvements in patient function. The technique appears to mitigate many of the risks associated with more invasive open procedures while providing effective fracture stabilization. However, larger prospective studies with extended follow-up are warranted to definitively establish the long-term safety profile of this technique and to identify potential risk factors for complications.

## Data Availability

The datasets generated and/or analyzed during the current study are not publicly available due patient confidentiality reasons but are available from the corresponding author on reasonable request.
